# How do patients with peripheral arterial disease communicate their knowledge about their illness and treatments? A qualitative descriptive study

**DOI:** 10.1186/s12912-016-0151-9

**Published:** 2016-05-04

**Authors:** Christine Wann-Hansson, Anne Wennick

**Affiliations:** Department of Care Science, Faculty of Health and Society, Malmö University, Jan Waldenströms gata 25, 20506 Malmö, Sweden; Vascular department, Skane University Hospital, Malmö, Sweden

**Keywords:** Peripheral arterial disease, Information, Focus group interviews, Nursing, Content analysis, Risk factor, Qualitative descriptions

## Abstract

**Background:**

Peripheral arterial disease is a chronic illness, and patients with peripheral arterial disease should receive advice about lifestyle changes and medical therapies to reduce further atherosclerotic complications. Previous research has indicated that patients with peripheral arterial disease lack information about their disease and secondary prevention measures. The aim was to elucidate how patients with peripheral arterial disease communicate their knowledge about their illness and treatments.

**Methods:**

During 2009, seven focus group interviews were conducted with twenty-one patients (50–81 years old) with peripheral arterial disease and were analysed using content analysis.

**Results:**

When respondents with PAD communicate their knowledge about the illness and its treatments they “*Navigate through uncertainty, believes and facts about PAD, displaying an active or passive information-seeking behaviour*”. After discharge, they felt a feeling of relief at first, which was later exchanged into uncertainty from their restricted knowledge about the illness and how to behave following revascularisation. For example, during the discussions about risk factors, smoking was noted as a major risk factor, that triggered feelings of guilt. Thus, the respondents needed to consult other sources of information to manage their everyday lives.

**Conclusions:**

Following endovascular treatment, the short amount of time spent with peripheral arterial disease patients requires innovative guidance in clinical practice to meet individuals’ needs regardless of whether the patient actively or passively understands and manages their peripheral arterial disease.

## Background

Peripheral arterial disease (PAD), defined by generalised atherosclerosis, has high cardiovascular and cerebrovascular morbidity as well as high mortality rates. The estimated prevalence is 3–10 %, increasing to 15–20 % in age groups over 70 [1]. PAD is a chronic illness, and the major goal of treatment is to provide symptom relief, improve functional ability and prevent the progression of generalised atherosclerosis [2]. Additionally, from symptoms ranging from intermittent claudication to rest pain to ulceration and gangrene, PAD is associated with several risk factors, such as age, diabetes, smoking, hypertension, dyslipidemia and physical inactivity [1]. Thus, patients with PAD are recommended to receive advice about lifestyle changes and medical therapies to reduce the risk of atherosclerotic complications. However, a previous review has shown that the majority of PAD patients do not receive complete information about secondary prevention measures [2]. Awareness of PAD symptoms, risk factors and treatment options are reportedly low in both the general population [3] and in patients with PAD [3–6]. However, few studies have explored how these patients actually communicate their knowledge about their illness and treatments.

Even though PAD is recognised as a chronic illness that cannot presently be cured, vascular surgery is an acute treatment in a cure-oriented field [2]. For example, a previous study has shown that patients believe that they are cured following revascularisation, which may delay their ability to adapt and adjust to the chronic disease [7, 8]. However, other qualitative studies have shown that patients with intermittent claudication are generally unaware of the causes of the disease and their increased risk of future cardiovascular health problems [8, 9]. The short hospital stay, rapid procedural technique and/or immediate relief of leg pain accompanied by increased functional ability may all be possible explanations for this belief [10]. Along with the advancement of endovascular procedures and shorter hospital stays [11, 12], the time available for education and information is decreasing, leading to a greater need for detailed information to enable patients effectively manage their care at home [13].

The relationship between literacy and health is another important aspect to consider when studying how patients communicate their knowledge about an illness and treatment. Health literacy is the degree to which individuals have “the capacity to obtain, process, and understand basic health information and services needed to make appropriate health decisions” [14]. This degree of literacy implies that patients may need more than a pamphlet and a health care appointment to incorporate necessary lifestyle changes. Furthermore, health literacy is critical to empowerment when patients’ access to health information and their capacities to use the information effectively are improved [15]. Therefore, it is important that patients are encouraged to participate in their own care via patient learning situations and individually tailored information [16]. From both the individual and societal perspective, patient participation can be defined as being involved in a life situation, including capacity and performance [17]. For the patient, being involved means having knowledge, being able to identify his/her own problems and interacting with health care professionals [18].

However, at present, knowledge about the possibilities for patients with PAD to participate in their own care, recover after revascularisation and prevent the progression of general vascular disease is scarce. In addition, long-term results after revascularisation have shown that many patients with PAD need lifelong treatment and that complementary efforts to prevent the progression of the disease are necessary [19, 20]. Thus, the aim of the study was to elucidate how patients with PAD communicate their knowledge about their illness and treatment.

## Methods

The study design was inductive and included focus group interviews with patients who were diagnosed with PAD. Focus group interviews were considered to be suitable, as the method emphasises the interaction between respondents with a common frame of reference [21]. This method allowed us an opportunity to elicit data not only about each individual’s experiences, but also about how he/she communicates with others. Content analysis was chosen as the methodological approach as previously described by Berg [22].

### Participants and setting

Using a purposive sampling technique*,* individuals diagnosed with PAD who had undergone a vascular intervention during the preceding six months at one vascular centre in Sweden were invited to participate in the study. The inclusion criteria were patients with PAD who were able to participate in focus group interviews and had undergone active vascular treatment. Of 51 eligible patients, 21 (nine males and 12 females) agreed to participate. Their mean age was 70 years (range 50–81 years). Data concerning risk factors were collected from patient medical records (Table 1). The vascular centre is located at a university hospital and treats approximately 300 patients with PAD annually*,* of whom a majority (75 %) undergoes endovascular interventions that include a one-day hospital stay [23].

**Table 1:** Demographic characteristics of the respondents

	All	Group 1	Group 2	Group 3	Group 4	Group 5	Group 6	Group 7
	*N* = 21	*n* = 4	*n* = 2	*n* = 3	*n* = 3	*n* = 4	*n* = 3	*n* = 2
Age m (range)	70 (50–81)	(71–80)	(71–72)	(64–75)	(62–76)	(61–81)	(58–75)	(50–60)
*Sex n (%)*								
Male/female	9 (42)/12 (58)	1/4	1/1	1/2	1/2	3/1	1/2	0/2
*Cohabitation n (%)*		^a^	^a^	^a^	^a^	^a^	^a^	^a^
Living alone	5 (24)							
Living with a significant other	16 (76)							
*Severity of the disease* *n (%)*								
Intermittent claudication	15 (71)	2	2	2	2	3	2	2
Ischaemia rest pain or ulcers	6 (29)	2		1	1	1	1	
*Risk factors n (%)*		^a^	^a^	^a^	^a^	^a^	^a^	^a^
Smoking	7 (33)							
Hypertension	13 (62)							
Heart disease	8 (38)							
Diabetes	5 (24)							
Stroke/TIA	1 (5)							
Kidney disease	1 (5)							
*Type of intervention n (%)*								
Endovascular	16 (76)	3	1	2	3	3	2	2
Open surgery	5 (24)	1	1	1		1	1	
*Previous vascular interventions n(%)* yes/no	9 (42/12 (58)	^a^	^a^	^a^	^a^	^a^	^a^	^a^

^a^Not applicable due to confidentiality

### Data collection

Seven focus group interviews were conducted between 2009 and 2010; each group consisted of 2–4 respondents (Table 1). To compensate for the small group sizes because some participants failed to turn up, seven instead of the recommended 3–5 focus group interviews were conducted to ensure saturation [24]. The interviews were conducted in a conference room at the hospital with the first author as moderator who had not previously met the respondents. The moderator started the interview by asking the respondents to narrate how they first noticed their leg problems and their perceived causes to give them an opportunity to become acquainted. A thematic interview guide was used by the moderator to keep the discussions on track, including areas such as *vascular disease* and *risk factors* as well as *treatment*. Additional questions were only asked to deepen or clarify the information. All interviews were digitally recorded, transcribed verbatim and lasted 60–120 min. The moderator made field-notes immediately after every interview, including a summary of the areas discussed, the order of speakers and group dynamics, which were reviewed to ensure the quality of each group session.

### Data analysis

The texts were analysed using manifest and latent content analysis influenced by Berg and Lune [22]. Manifest content analysis was employed to elicit the perspective of the respondents’ descriptions, which was physically present in the text. Latent content analysis was used to capture the deep structural meaning of the text as well as to illuminate the meaning of illness and treatment among patients diagnosed with PAD. Initially, both authors independently read through the transcripts in order to understand each interview as a whole (naïve reading). Words or phrases carrying a meaning of importance for the aim of the study, so-called meaning units, were subsequently identified [25]. Each meaning unit was evaluated by the following questions: “what is it about?”, “what does it mean?” and “what effect does it have?” (Table 2). Emerging meaning units were coded on the basis of the content in order to identify patterns of similar phrases, relationships and commonalities or disparities. The codes were critically analysed and grouped into categories. Finally, the authors discussed and reflected on the findings, and taking their pre-understanding and the research question into account. Four categories were disclosed and when combined into an interpreted whole, a main theme emerged.

**Table 2:** Examples of meaning units, interpreted meanings and categories

Meaning units	What is it about?	What does it mean	What effect does it have?	Categories
”..that I didn’t know but found out was that it is senile decay and you can’t take it away like you do when you clean a coffee maker (everyone laughs) it becomes permanent and then they have to go inside the critical parts of the vessel and expand it…..Then it should work again. (Liza, Group 7)”	Descriptions of peripheral arterial disease	A need to understand the atherosclerotic process and the vascular treatment by using own words	Leading to a believe of how the atherosclerotic process and the vascular treatment function	Describing the known and unknown
”I didn’t believe that smoking was…..but the doctors and nurses told me that smoking was the greatest villain” (Judith Group 6).	The risk of smoking and it’s harmful effects	A wish to repress the risk of smoking and it’s harmful effects	A possible first step in considering giving up smoking	Conflicting feelings towards smoking
”But in some cases it would help if you could all calm down a little when you are talking to us. I have met doctors who say hmhmhm and you notice that they are stressed and then you have no further questions…..Once I met a doctor who was outside the door before he had finished speaking and I heard the last word from outside*”* (Frank, Group 2).	The experience of meeting health care professionals who suffer from stress	In an attempt not to disturb the patients is left with unanswered questions	A need to have face to face access to health care professionals	Consulting various sources of information
When I came home I didn‟t know what to do, so when a week had passed I thought – I have to go to the vascular clinic!, because I didn‟t know if I dared to take a bath, as I had plasters which I was afraid to remove myself. They were all bloody…‟(Marilyn, Group 1).	Descriptions of uncertainty when being back home again.	Lack of discharge information	Feelings of uncertainty and insecurity	Feeling relieved, yet uncertain

## Results

When respondents with PAD communicate their knowledge about the illness and its treatments they “*Navigate through uncertainty, believes and facts about PAD, displaying an active or passive information-seeking behaviour*”. Descriptions about PAD, treatments and risk factors associated with atherosclerosis pended between believes and facts. This formed the first category; *describing the known and unknown.* Smoking was, however, a well-known risk factor that triggered feelings of guilt and ambivalence due to its harmful effect, which formed the second category; *conflicting feelings towards smoking*. Initially after treatment, the respondents felt relieved, a feeling that was gradually overshadowed of uncertainty. This was a result of their restricted knowledge about both the illness and how to behave following revascularisation, which formed the third category; *feeling relieved, yet uncertain*. Thus, the respondents with regard to their information seeking behaviour need to consult other sources of information to manage their everyday life, which formed the last category; *consulting various sources of information.*

The findings revealed a use of different strategies (active or passive) when seeking information that had an influence on how the respondents communicated their illness and treatment. Individuals with active information-seeking behaviours used different sources to fulfil their needs and frequently asked questions when meeting with health care professionals. Individuals with passive behaviours did not experience the same urge to ask questions concerning their disease nor did they want such detailed information as those with active behaviour. Quotations from the original text, marked with fictive names, are presented below to emphasise its inherent meaning.

### Describing the known and unknown

In general, the respondents felt they had insufficient knowledge about cardiovascular diseases to fully understand the aetiology, consequences, risk factors and preventive options of their illness. The respondents initially did not know the cause of their leg symptoms but thought that time would relieve the pain and growing old was considered to be the main explanation. It was also unclear whether the respondents really understood their medical procedures. Yet everyone described the treatment procedure in detail regardless of whether it was open surgery or an endovascular intervention. Respondents who were active information-seekers frequently used medical terminology when describing their experiences by comparison those with a more passive approach had fewer definite beliefs about the disease and treatments. Their descriptions were often based on self-invented words, such as the following explanation expressed by one man: *“…Once they told me that is a Chicken wire… that they put in a wire. How can they do that? I don’t know what kind of wire that is, if it is plastic?…”* (David, group 4).

Respondents with passive information-seeking behaviour had a more fatalistic view and were unaware of the chronic nature of PAD or the significance of lifestyle changes; by contrast, those with more active information-seeking behaviour were relatively well informed about their vascular disease. An example of these different approaches is illustrated by the focus group discussion below:

Bertha: *“…I am that type of person. I don’t ask much nor do I want to know too much…”*.

Judith: *“…I want to know. I want clear information so that I know what to expect…”*.

Charles: *“…Right, I want to be able to prevent and change things. If they had said, - You only have four months left now! Then I would have to do a lot to be able to do everything that I want…”*.

Bertha: *“…I am not like that at all…”* (group 6).

In contrast to respondents with passive information-seeking behaviour, the active respondents knew that PAD is a general disease that affects all blood vessels in the body and were aware of the importance of lifestyle changes. References were frequently made to the importance of a healthy diet due to its effects on cholesterol. Genetic characteristics were also a well-known reason for PAD and described as being beyond their control.

### Conflicting feelings towards smoking

Smoking was discussed in all sessions. Those who were smokers or ex*-*smokers experienced feelings of guilt that varied from regretting ever being a smoker to denying the harmful effects of smoking. Those who had quit smoking expressed a strong need for confirmation and encouragement to maintain their non-smoking status. Starting to smoke again was explained as a result of a return of the PAD symptoms, which lowered the motivation to remain a non-smoker. Another reason was a feeling of regained health, reducing the fear of smoking. It would be more motivated to quit smoking if the diagnose instead was myocardial infarction or cancer. One man said: *“…Mostly you hear of the risk of lung cancer, even if the risk of circulatory and heart diseases is actually higher, but lung cancer is somehow more frightening than a vascular disease…”* (John, group 4).

Being told by a physician to quit smoking had some effect but could also be experienced as an order and a personal insult*,* regardless of its impact on their illness. Being a smoker with an active information-seeking behaviour meant having knowledge about the harmful effects of smoking and the whole catalogue of available aids to quit. However, having knowledge did not always equate with non-smoking.

### Feeling relieved, yet uncertain

The respondents discussed their need for knowledge when returning home after treatment. At first, the respondents experienced feelings of relief after have been discharged. They were overjoyed with the almost immediate improvement in their legs and content with the advice to return home and live their lives as usual. It was not until after a few days at home that further questions arose about stitches and wounds, whether showering was allowed, how far it was recommended to walk, the extent to which they could do strenuous work and for how long they would have to take medication. Another question was how long the stents lasted and whether they could become loose. Respondents with active information-seeking behaviour contacted the hospital for additional information. One female said: “*…When I came home I didn’t know what to do, so when a week had passed I thought – I have to go to the vascular clinic! Because I didn’t know if I dared to take a bath, as I had plasters which I was afraid to remove myself. They were all bloody…‟* (Marilyn, group 1).

Those with active information-seeking behaviour worried about the risk of being stricken by a myocardial infarction or stroke, whereas respondents with passive information seeking behaviour tried to retain a more positive view of the future. Their philosophy was *“…what you don’t know won’t hurt you”.* First-time patients were also afraid that their pain would return, while those who had previous vascular interventions knew that the risk existed. As one female said: *“…I do a lot of walking because I want to walk. I have stopped for two weeks now, which is not such a very long time, but the walks may very well end any time…‟*(Inga, group 2).

### Consulting various sources of information

During the discussions, the respondents described the different ways they were using to obtain knowledge about PAD. The most valuable source of information was the physician although nurses were also appreciated. However, respondents experienced a lack of face-to-face meetings with health care professionals and the physician was often seen to suffer from stress. Thus, the most common procedure at discharge was a brief meeting with the physician sitting on the edge of the bed. The respondents could not recall receiving any specific discharge information. Consequently, the respondents often kept their questions to themselves in order not to disturb the health care professionals. As one woman stated: *“…I missed the interaction with the doctor…or with a nurse at least…”* (Liza, group 7).

Other sources used to gain knowledge were from TV, newspapers and friends or family members. The respondents reflected together on different ways to fulfil their need for knowledge such as information leaflets, including detailed information about the treatment and pictures illustrating the procedure they had undergone. This type of information was requested both before the hospital stay and at discharge. As one man said: *“…I would have appreciated seeing a picture, showing how it is done and how it works, then I would have known what it is like…”* (Charles, group 6).

The respondents, regardless of active or passive information-seeking behaviour, expressed these needs. They also requested pedagogical TV programmes to be shown on the ward in order to optimise their hospital stay. The rationale behind this suggestion was that patients have plenty of time during their hospitalisation to prepare themselves before a procedure or an appointment with the doctor, as one woman has expressed: “…*It might be good to receive some of the information during the hospital stay because you have to wait quite a lot and you need something to distract you…”* (Judy, group 5). DVDs or computers for domestic use were not an option, as the respondents considered themselves to be too old for such equipment.

## Discussion

Knowledge about peripheral arterial disease and treatment was communicated by navigating through uncertainty, beliefs and facts and varied from respondent to respondent. Whereas some actively sought information and were relatively well-informed, others had a more fatalistic view to life and found it not as important to have further knowledge about their illness. These various information-seeking behaviours and needs for knowledge may have several explanations. For instance, a person’s health literacy may affect both his/her information-seeking behaviour and recall of information [26, 27]. Differences in demographics, personal experiences, salience and beliefs are other factors affecting a person’s underlying motivation to seek information [28]. Thus, information and education programmes provided by health care professionals have to be adjusted to suit every patient’s profile as well as his or her individual needs.

The respondents were aware of the risk of smoking and high cholesterol. However, besides that, their understanding of risk factors was low; for example, hypertension, diabetes and inactivity were not mentioned. This finding corresponds with previous studies regarding awareness of vascular disease risk factors. Smoking and high cholesterol have also been reported as the most identified risk factors among patients with coronary heart disease [29] and PAD [3, 4, 9]. This finding may be due to the relatively aggressive national and international advertising campaigns against smoking and high cholesterol. In our respondents, awareness of these risk factors was, however, not the same as having knowledge about how to reduce the risk because several of the respondents continued to smoke despite knowing about the harmful effects. Endovascular procedures and the short hospital stay may deceive the patients into believing that they are cured and, thus, do not need lifestyle changes. Hence, the findings highlight a need for ways to further inform patients about risk factor prevention.

The respondents return home after revascularisation was at first associated with a feeling of relief, but after a couple of days, this was replaced by feelings of uncertainty. This finding has also been seen in surgical patients who did not receive necessary information, implying a risk of problems after discharge [16, 30]. As found in other studies [31, 32], the most common questions after discharge concerned hygiene, wound care, stitches and activity. Thus, patients who leave the hospital with little knowledge may not confidently manage their condition and may therefore seek help from health care services [16, 33]. Furthermore, particular medical words or phrases must be used more carefully by health care professionals to increase the understanding and the possibility to recall information [26]. Our respondents with active information-seeking behaviour, who post-operatively contacted the hospital more often than their passive counterparts, represent a patient group who will manage, despite having received inadequate discharge information. It is therefore essential to identify persons with a more passive approach and determine what type and amount of information the individual patient needs before discharge. Preferably, relatives or significant others should also be present and notified about the discharge information.

Although varied sources of knowledge was communicated and utilised, face-to-face meetings with health care professionals were preferable, and the lack thereof was experienced as a shortcoming in the exchange of knowledge. Lack of time, deficits in verbal information and the fact that written information is less helpful than face-to-face information has also been stressed in other studies [34–36]. According to the Comprehensive Model of Information Seeking, an individual often uses both interpersonal and mediated channels to obtain information. The interpersonal channel involves health care professionals, family members as well as close friends [28]. It also reflects the most common source of knowledge communicated by the respondents in the present study whereas media channels, such as the media and the internet, was not equally appreciated. Previous studies have, however, shown that web-based education and information are important to increase patients’ knowledge about their condition [37] and increase empowerment [38]. Thus, technology, such as the internet, must be seen as complementary and an area for further development as patients feel more comfortable using computers and the Internet.

The findings suggest that the respondents did not actively participate in their care. It seemed that they often stood back and did not want to bother the health care professionals with their questions. Hence, they were neither informed enough to participate in their own care nor invited to do so. Although research has shown that knowledge and education are essential to facilitate patient participation [18, 39], health care professionals tend to assume the role of an expert and authority without offering adequate resources for the patient to become an active participant [40]. Time is another critical factor because it is required for collaboration between health care professionals and patients and has been considered to be a barrier to patient participation [41]. A further demonstrated barrier is how the care is organised and planned when patients, during their hospitalisation, have to meet new faces all of the time [40]. This finding implies that the short hospitalisation following endovascular treatment requires new innovative guidance to ensure sufficient time for knowledge-sharing between patients and health care professionals.

### Study limitations

Some of the limitations of focus group methodology deserve attention when interpreting the findings of this study. For example, group discussions can lead to group consensus, which may influence the creation of particular ideas [42]. To minimise this risk, the moderator encouraged participation by less vocal members. The ideal group size has been debated, and there is little consensus about what is appropriate. A review of the literature indicated that the number of participants can vary from 4–20 [21]. Because the sample size in some of the groups in the present study was low, there may also be a risk of fewer generated concepts [21]. Fewer people in a group may, however, increase the likelihood of interaction [42], and because several of the respondents were older and had impaired hearing, small groups were preferable. To increase the credibility of the study, all discussions were held at the same location. The focus group interviews were performed within six months postoperatively and had, thus, a retrospectively approach. Although the time lap varied, all respondents easily went back to their previous experiences related to their illness and treatment.

### Recommendations for practice

Following vascular interventions, patients seem to lack knowledge and education about general atherosclerotic disease as well as post-surgery procedures. Thus, discharge information and education have to be both structured and individualised, which can be supported by tailored educational materials. However, oral communication in daily practice is irreplaceable, and health care professionals need to learn about a patient’s views and expectations in order to facilitate participation. Nurse-led follow-up programmes can be an effective way to give patients the competence to make decisions and enable them to participate more fully in their own care. A potentially important motivator is the use of self-assessment regarding PAD and knowledge about risk factors, which allows identification of misconceptions and knowledge gaps. For a new generation of patients, greater emphasis should be placed on the diversity of educational methods, especially the use of modern technology for an independent study.

## Conclusion

How patients with peripheral arterial disease (PAD) communicate their knowledge about the illness and treatment was influenced by their information-seeking behaviour. They navigated through uncertainty, beliefs and facts about their illness and treatment with a discernible need of further knowledge about vascular disease and strategies for risk factor prevention. The short time spent with patients following endovascular treatment requires evidenced-based and innovative guidance in clinical practice in order to meet individual needs. It is, however, important to consider different aspects of health literacy and how it may affect the patient’s choice in actively or passively participating in the understanding and management of PAD. Hence, further research should focus on the implementation and evaluation of structured education programmes, perhaps supported by modern technology as a complement to oral communication. This strategy may facilitate patients’ ability to participate and be involved in their own health and treatment.

### Ethics and consent to participate

A nurse responsible for coordinating vascular interventions sent an informational letter to eligible patients. The information letter included an invitation to participate in the study stated that they would be contacted by phone, within a week. Thereafter, they were contacted by telephone by the first author and provided with oral information, emphasising the voluntary nature of the study and that all data would be treated confidentially. Information about the need to collect data from the patients’ medical record was also emphasised. After being given time to reflect, those who agreed to participate were scheduled for an interview. Written informed consent was obtained in conjunction with the interview. Ethical approval was granted by The Regional Ethics Committee (No. 315/2008).

### Consent to publish

Not applicable.

### Availability of data and materials

Data will not be shared due to confidentiality.

## Abbreviations

PAD: Peripheral arterial disease
